# Characterization of the complete chloroplast genome of the *Solanum tuberosum* L. cv. Shepody (Solanaceae)

**DOI:** 10.1080/23802359.2021.1934135

**Published:** 2021-07-14

**Authors:** Shanshan Chen, Yanfei Zhao, Jing-Ying Zhang, Jia-Yue Zhang, Ya-Ping Wang, Bin Mou, Haoran Ma, Zhijun Han, Yue Lu, Shuang Li, Chun-Bo Zhao, Yu Zhu Han

**Affiliations:** College of Horticulture, Jilin Agricultural University, Changchun City, P.R. China

**Keywords:** *Solanum tuberosum*, complete chloroplast genome, phylogenetic analysis, Solanaceae

## Abstract

Potato (*Solanum tuberosum* L.), a species of the family Solanaceae, is the fourth most important food crop worldwide. *Solanum tuberosum* L. cv. Shepody is a long, smooth, white-skinned potato cultivar with medium green leaves. It has good specific gravity and boils and bakes well. To support more molecular data for breeding of S. tuberosum, the complete chloroplast (cp) genome sequence of *S. tuberosum* L. cv. Shepody was determined using the next-generation sequencing. In leaves, the chloroplast genome accounts for 3.88% of the total genome. The entire cp genome was determined to be 155,296 bp in length. It contained large single-copy (LSC) and small single-copy (SSC) regions of 85,737 and 18,373 bp, respectively, which were separated by a pair of 25,593 bp inverted repeat (IR) regions. The genome contained 132 total genes, including 87 protein-coding genes, 37 tRNA genes, and 8 rRNA genes. The overall GC content of the genome is 37.9%. A phylogenetic tree reconstructed by 60 chloroplast genomes reveals that *S. tuberosum* L. cv. Shepody was closely related to S. tuberosum L. cv. Desiree with bootstrap support values of 100%.

*Solanum tuberosum* L. (family: Solanaceae) has high nutritional value, adaptability, and large yield. It is the largest non-cereal food crop worldwide and ranked as the world's fourth most important food crop after rice, wheat, and maize (Horton and Sawyer 1985; Zhang et al. 2017). *Solanum tuberosum* L. cv. Shepody (https://www.europotato.org/varieties/view/Shepody-E) is a long, smooth, white-skinned potato cultivar with medium green leaves. It has good specific gravity and boils and bakes well (Young et al. 1983). Since the main sites of starch synthesis are amyloid and chloroplast, and the chloroplast genome contains many genes involved in starch synthesis, it is necessary to characterize the chloroplast genome of the potato Shepody.

Healthy leaf samples were collected from a tissue culture plant (E:125.417353, N43.821995). The total genomic DNA was extracted from the fresh leaves of *S. tuberosum* L. cv. Shepody using the DNeasy Plant Mini Kit (Qiagen, Valencia, CA) and stored in College of Vegetable Science, Jilin Agricultural University (JAUSBD01). After DNA isolation, 1 μg of purified DNA was fragmented and used to construct short-insert libraries (insert size ∼350 bp) according to the manufacturer’s instructions (BGISEQ) detailed in the previous literature (Huang et al. 2017). Then DNA libraries were sequenced by Hefei Bio&Data Biotechnologies Inc. (Hefei, China) on the BGISEQ-500 platform with PE150 read lengths. The filtered reads were assembled using the program NOVOPlasty Version 3.8.3 **(**Dierckxsens et al. 2017). The cp-genome was annotated with the GeSeq (Tillich et al. 2017) and tRNAscan (Schattner et al. 2005).

In leaves of *Solanum tuberosum* L. cv. Shepody, the chloroplast genome accounts for 3.88% of the total genome, which may have contributed to its medium green leaves and relatively high starch production. The chloroplast genome was determined to comprise double-stranded, circular DNA of 155,296 bp containing two inverted repeat (IR) regions of 25,593 bp each, separated by large single-copy (LSC) and small single-copy (SSC) regions of 85,737 and 18,373 bp, respectively (Genbank acc. no. MW307949). The genome contained 132 total genes, including 87 protein-coding genes, 37 tRNA genes, and 8 rRNA genes. Seven protein-coding genes, six tRNA genes, and four rRNA genes were duplicated in IR regions. Nineteen genes contained two exons and four genes (clpP and ycf3 and two rps12) contained three exons. The overall GC content of *S. tuberosum* L. cv. Shepody cp genome is 37.9% and the corresponding values in LSC, SSC, and IR regions are 36.0, 32.1, and 43.1%, respectively. Heteroplasmy testing showed that there are about 181 low-frequency SNP sites with minor allele frequency (MAF) ≥0.03 and ≥5× reads coverage in the chloroplast genome of potato Shepody. Most of these SNPs are located between ycf15 and trnL-CAA.

To investigate its taxonomic status, an ML tree was reconstructed based on whole chloroplast genomes from 59 *Solanum* plants and one outgroup plant (*Nicandra physalodes*) by Mafft version 1.4 and FastTree version 2.1.11 with Generalized Time-Reversible (GTR) model, 1000 bootstrap replicates (Price 2010). The ML phylogenetic tree shows that *S. tuberosum* L. cv. Shepody was closely related to *S. tuberosum* L. cv. Desiree with bootstrap support values of 100%. Furthermore, the chloroplast resource still will provide molecular genetic information for DNA barcoding, conservation genetics, and breeding of *S. tuberosum* in the future (Figure 1).

**Figure 1.**
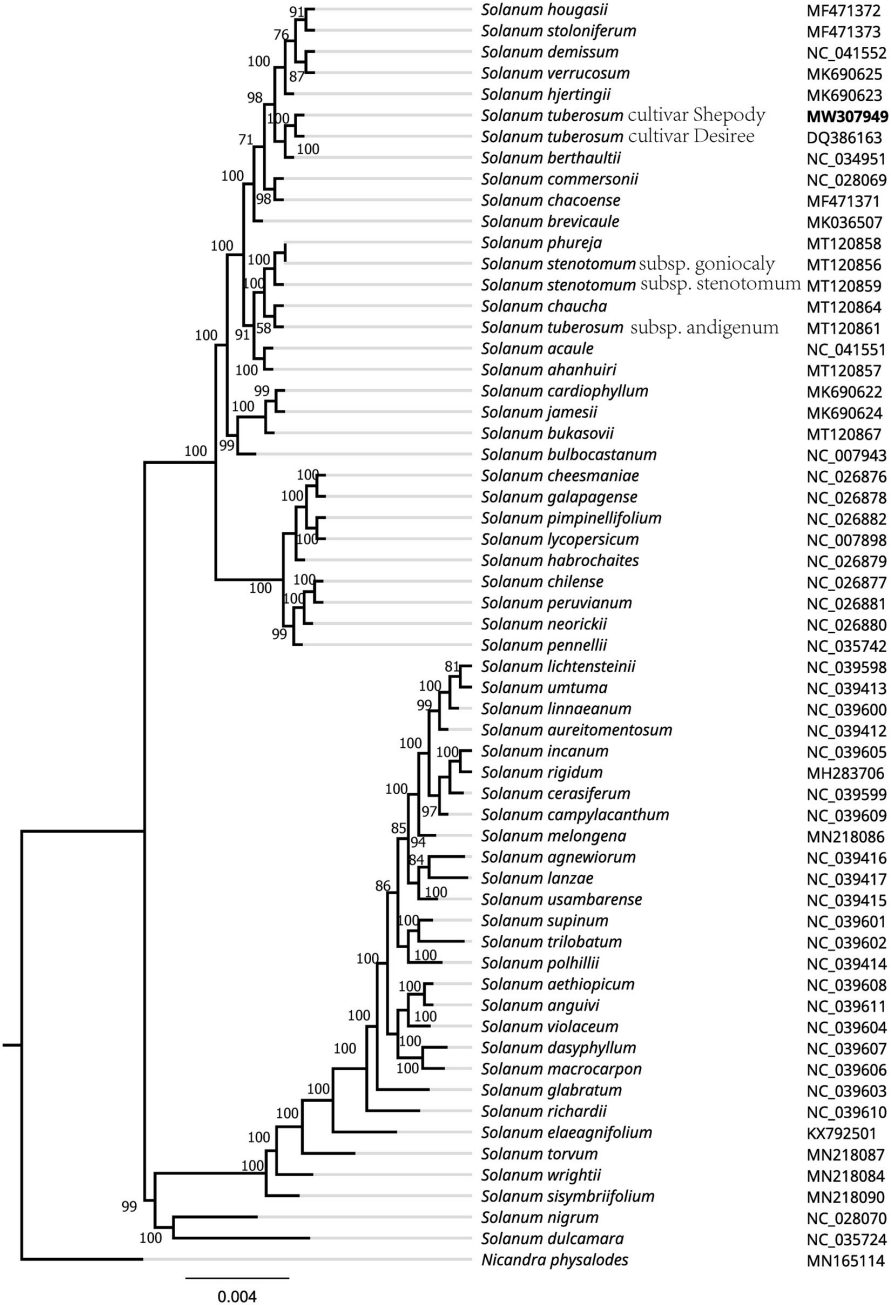
Maximum-likelihood phylogenetic tree based on whole chloroplast genomes from 60 *Solanum* plants and one outgroup plant (*Nicandra physalodes*) and the support values are shown at the branches.

## Data Availability

The complete chloroplast genome sequence of *Solanum tuberosum* L. cv. Shepody is deposited in the Genbank (https://www.ncbi.nlm.nih.gov/search/all/?term=MW307949) database under the Accession no. MW307949. The raw sequencing data are deposited in the SRA database (https://trace.ncbi.nlm.nih.gov/Traces/sra/?run=SRR13162918) under the Accession no. SRR13162918.
